# Conservative Management of Acute Nontraumatic Mediastinitis in Children: A Case Series

**DOI:** 10.1155/crpe/3863809

**Published:** 2026-08-17

**Authors:** Stacy Schertenleib, Céline Callias, Pierre Alex Crisinel, Margherita Plebani

**Affiliations:** ^1^ Faculty of Biology and Medicine, University of Lausanne, Rue du Bugnon 21, Lausanne 1011, Switzerland, unil.ch; ^2^ Department of Pediatrics, Réseau Hospitalier Neuchâtelois, Pourtalès Maladière 45, Neuchâtel 2000, Switzerland; ^3^ Women-Mother-Child Department, Unit of Pediatric Infectious Diseases and Vaccinology, Service of Pediatrics, Lausanne University Hospital and University of Lausanne, Rue du Bugnon 50, Lausanne 1011, Switzerland, chuv.ch

**Keywords:** acute nontraumatic mediastinitis, case series, conservative management, descending necrotizing mediastinitis, pediatric

## Abstract

Acute nontraumatic mediastinitis is a rare and potentially life‐threatening condition. In children, management strategies remain poorly standardized. Here, we describe four pediatric cases: two originating from cervical infections (descending necrotizing mediastinitis [DNM]) and two with indeterminate source. The first two patients presented with febrile otorhinolaryngologic symptoms, one complicated by secondary respiratory failure and the other by possibly toxin‐related symptoms. The third presented with varicella and scarlet fever‐like rash, complicated by multiorgan involvement secondary to a probable toxic shock syndrome. The fourth case presented with fever without focus and shoulder pain. Three patients developed secondary pleural effusions. In three cases, diagnosis was confirmed by computed tomography (CT) scan and in one case by MRI. None had necessitated surgical drainage. All patients were managed conservatively with prolonged antibiotic therapy and achieved complete recovery without sequelae. These cases suggest that, in selected pediatric patients, a conservative, antibiotic‐based approach can be both safe and effective.

## 1. Introduction

Acute nontraumatic mediastinitis in children is a rare but severe infection. Its management is not standardized and relies mainly on data from adult populations [1].

The mediastinum is the intrathoracic anatomical region bordered by the mediastinal pleura, the thoracic inlet, and the diaphragm. The majority of pediatric mediastinitis is of “traumatic” origin, caused by esophageal injury (foreign body ingestion) or cardiothoracic surgery. Acute nontraumatic mediastinitis is much rarer, and diagnosis and treatment can be challenging. The most clearly defined entity is descending necrotizing mediastinitis (DNM). It results from a retropharyngeal infection spreading to the upper mediastinum. The retropharyngeal space contains lymph nodes, which typically regress by the age of 5 years, explaining the decline in retropharyngeal abscesses and related infections after this age. However, persistent lymphatic tissue or contiguous spread from a nearby infection may still lead to infection after the age of 5 years [2, 3]. Posterior to the retropharyngeal space lies the alar fascia. If the retropharyngeal abscess breaches the alar fascia, the bacteria can enter the “danger space” (delimited by the alar fascia and the prevertebral division of the deep layer of the deep cervical fascia and extended from the skull base and the diaphragm) and potentially spread to mediastinum [4, 5]. Other, less common pathways of mediastinal infection include contiguous spread (from pleuropulmonary, bone, or soft‐tissue infections of the thoracic cage) and, rarely, hematogenous dissemination. In the pediatric population, the most frequently identified microorganisms responsible for this condition are *Streptococcus pyogenes* and *Staphylococcus aureus* [6, 7].

A contrast‐enhanced CT scan of the neck and chest is recommended to establish the diagnosis [7–9], even though radiation exposure remains a concern in children. Diagnosis may be difficult because young patients often struggle to describe their symptoms [10] and because clinical presentations are diverse and frequently nonspecific [9]. Moreover, as with other severe pediatric bacterial infections, DNM may follow or coexist with a viral illness, making early diagnosis challenging as initial symptoms may be mistakenly attributed to a self‐limiting viral infection rather than an underlying bacterial process.

Pediatric literature is less extensive than that of the adult population and consists solely of case reports and small case series [1, 6, 7, 9–11]. Deu‐Martin et al. estimated the annual incidence of DNM among adults at 5.1 cases per million inhabitants [12]. The frequency of this condition is difficult to estimate in children. However, its frequency can be approximated based on the proportion of deep neck abscesses that progress to DNM. Adil et al. reported an incidence of pediatric deep neck space infection of 4.6 per 100,000 children in 2009 [13]. In a series of 100 pediatric deep neck abscess cases described by Patigaroo et al., two were complicated by mediastinitis [14]. Applying this proportion to the incidence reported by Adil et al. suggests an estimated incidence of approximately 9 cases of mediastinitis per 10,000,000 children.

In adults, reported mortality rates are very high (15%–40%) [1, 15, 16], and early, aggressive surgical management appears to improve outcomes [7, 17]. Pediatric mortality appears lower (< 10%) [1, 8], but the rarity of this condition leads to a risk of delayed diagnosis [7]. Once the diagnosis is established, the subsequent management remains matter of debate. The rapid initiation of antibiotics targeting pathogens of deep neck infections, and including MRSA coverage in high‐incidence settings, is clearly recommended [1, 9]; however, the optimal duration of antibiotic therapy and the added value of surgical intervention remain poorly defined [18].

We report four cases of acute nontraumatic pediatric mediastinitis that occurred in our tertiary‐care center over the last four years (between December 2022 and February 2025) and that were all successfully treated without surgery.

## 2. Methods

The “Joanna Briggs Institute Critical Appraisal Checklist for Case Reports” was respected for the redaction of our case series [19].

### 2.1. Case Presentations

#### 2.1.1. Case Report 1

**FIGURE 1:**
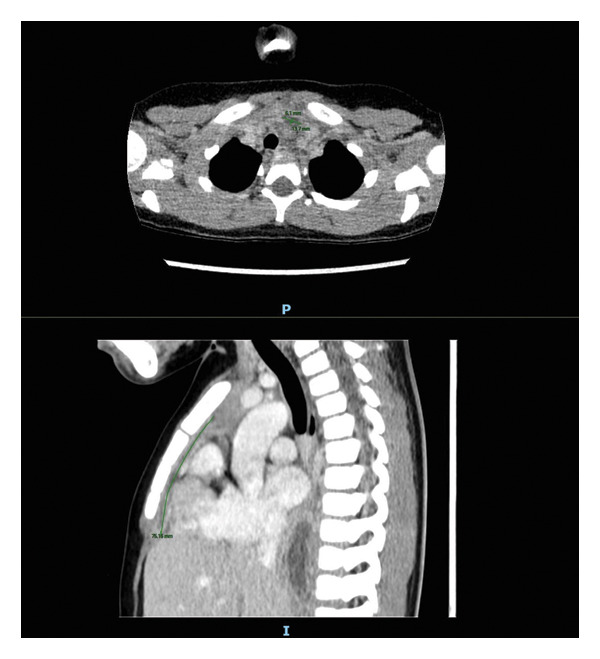
CT image from Case 1 showing the mediastinal fluid collection. The superimposed green lines indicate its maximum long‐ and short‐axis dimensions. P, posterior; I, inferior.

The patient was a 6‐year‐old girl, fully vaccinated and with no significant past medical history. She presented with a 48‐h history of fever, torticollis, odynophagia, left lower cervical pain, abdominal pain, and vomiting. On clinical examination, she had nonexudative pharyngitis, a heart rate (HR) of 134/min, a respiratory rate (RR) of 54/min, a temperature of 37.1°C, and an oxygen saturation (SpO_2_) of 95% on room air. Laboratory tests showed a C‐reactive protein (CRP) level greater than 200 mg/L and a white blood cell (WBC) count of 13.4 × 10^9^/L with neutrophilia. A contrast‐enhanced CT scan demonstrated diffuse infiltration of the left anterior cervical fat and anterior mediastinum, with a small (75 × 6 × 14 mm), nonorganized, nonencapsulated fluid collection extending superiorly from the level of the thyroid isthmus anteriorly to the latter, in front of the thymus and behind the sternum along its entire length (Figure 1). The patient was thus diagnosed with DNM, and after multidisciplinary discussion, the collection was not considered amenable to surgical drainage.

The patient was treated with intravenous amoxicillin–clavulanate (150 mg/kg/day). On hospital Day 3, she developed acute respiratory failure, and CT scan revealed bilateral pleural effusions and stable mediastinal involvement but new pulmonary foci. She required noninvasive ventilation (NIV) for 72 h and placement of a left‐sided pigtail pleural drain, which yielded purulent fluid and was removed 3 days later. Despite clinical deterioration, antibiotic therapy was continued unchanged, as amoxicillin–clavulanate was considered to provide adequate coverage for an ENT‐origin infection in our local epidemiological context. Blood and pleural fluid cultures were sterile, and pleural fluid pathogen‐specific real‐time PCR assays for *Staphylococcus aureus* and *Streptococcus pneumoniae* were negative. Broad‐range bacterial 16S rRNA gene PCR followed by Sanger sequencing of the amplicon identified *Streptococcus pyogenes*, showing 99% sequence identity (584/586 nucleotides) by NCBI BLAST analysis (BLAST, https://blast.ncbi.nlm.nih.gov/Blast.cgi). Her clinical, laboratory, and radiological status progressively improved, allowing a switch to oral amoxicillin (80 mg/kg/day) on hospital Day 7.

On hospital Day 10, worsening inflammatory markers led to a repeat CT, which demonstrated organization and consolidation of the empyema. Antibiotic therapy was not modified, given the high susceptibility of *S. pyogenes* to amoxicillin and the tendency of this pathogen, in our clinical experience, to cause highly inflammatory presentations with a prolonged course. Fever resolved on hospital Day 13.

The patient was discharged home 2 weeks after admission, with a total antibiotic course of 28 days. At the end of therapy, she had mild exertional dyspnea, and chest radiography showed residual pleural thickening. She was completely asymptomatic 10 months later. An immunological work‐up was performed and did not reveal any underlying immune deficiency.

#### 2.1.2. Case Report 2

The patient was an 8‐month‐old boy, fully vaccinated and with no significant past medical history. He was referred by a regional hospital for suspected severe viral croup with acute respiratory failure, after 4 days of cough, one of stridor, and a maximum temperature of 40°C. On arrival, his RR was 36/min, HR 184/min, temperature 38.2°C, and SpO_2_ 98% with 40% FiO_2_ (high‐flow oxygen 15 L/min). Laboratory tests revealed a CRP level of 61 mg/L and a WBC count of 4.8 × 10^9^/L without neutrophilia. A nasopharyngeal viral PCR was positive for adenovirus and picornavirus (rhinovirus or enterovirus). He received continuous adrenaline nebulization, NIV (6 days) and systemic corticosteroids (8 days).

**FIGURE 2:**
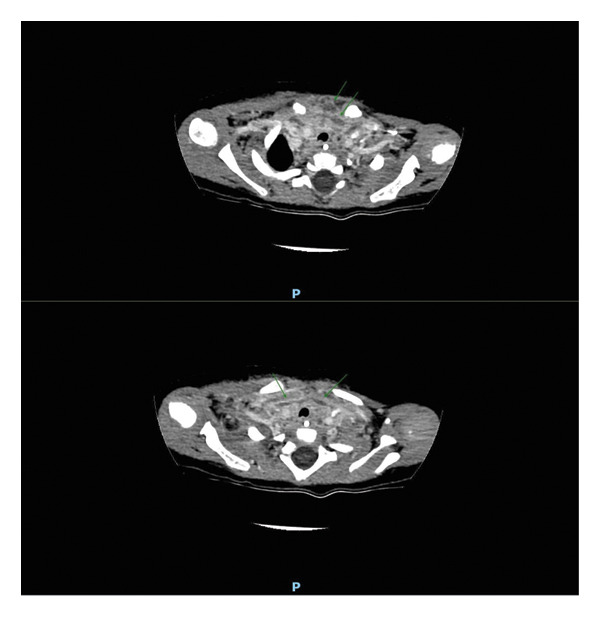
CT image from Case 2 obtained on day 9, showing multiple poorly defined mediastinal fluid collections indicated by the green arrows. P, posterior.

On Day 2, he developed a diffuse rash and CRP increased to 228 mg/L. A CT scan revealed a DNM with phlegmonous infiltration of deep cervical space, extending from retropharyngeal space to the upper mediastinum. He received one dose of ceftriaxone and was then treated with IV amoxicillin–clavulanate (150 mg/kg/day) and clindamycin (stopped at Day 5). His clinical condition improved, but from Day 8, he developed recurrent fever and inflammatory flare‐ups (CRP increase). A repeat CT scan (Day 9) showed several poorly defined collections consistent with phlegmonous areas in the anterior mediastinum and left paraoesophageal region, a new pleural effusion and a left pulmonary consolidation (Figure 2). Given recurrent rashes with cervical and thoracic swelling suggestive of a toxic component, clindamycin was reintroduced (Day 10–25) and corticosteroids (2 mg/kg) were restarted on Day 11 for 7 days. On Day 18, glove‐and‐finger desquamation of the hands and feet was observed. *Streptococcus pyogenes* infection was suspected based on the clinical course and the contemporaneous epidemiology of 2022–2023, but blood cultures showed no growth. He finally became afebrile on Day 28 and was discharged on Day 30, asymptomatic, with oral amoxicillin–clavulanate (80 mg/kg/day) for a total of 8 weeks.

Three days after stopping antibiotics, the patient was readmitted with fever, rhinorrhea, and conjunctivitis (CRP 125 mg/L). A viral infection was suspected, and a nasal swab PCR was positive for a picornavirus. MRI showed a suppurating left cervical lymph node, a right paratracheal nodal mass, and a right supraclavicular tissue infiltrate. Ultrasound‐guided aspiration was noncontributive. He was treated with a further 7‐day course of oral amoxicillin–clavulanate and discharged on Day 2, afebrile and asymptomatic.

He remained well at 1‐month follow‐up, and immunological work‐up to rule out immunodeficiency was normal.

#### 2.1.3. Case Report 3

The patient was a 6‐year‐old boy, fully vaccinated with no significant past medical history. He was admitted to a regional hospital with fever up to 40°C for 4 days, 48 h of “flu‐like” symptoms, varicella‐ and scarlet fever‐like rashes, abdominal pain, vomiting, and odynodysphagia. On arrival, he was afebrile with a HR of 120/min. Examination showed an erythematous pharynx without exudate, intermittent grunting with normal respiratory auscultation, and a tender abdomen without peritonism. Laboratory tests revealed a CRP of 198 mg/L, WBC of 11.5 × 10^9^/L with neutrophilia, and lactate of 3.4 mmol/L, with no signs of organ dysfunction. The rapid group A *Streptococcus* (GAS) test was positive. Fear of invasive *S. pyogenes* infection following varicella led to hospitalization and treatment with IV amoxicillin–clavulanate (150 mg/kg/day).

On Day 2, he developed signs of shock (HR of 140/min and BP of 84/50 mmHg) and multiorgan involvement (AST/ALT 2x the normal value, respiratory insufficiency, proBNP of 2052 ng/L, signs suggestive of disseminated intravascular coagulation (TP 58%, PTT 67, and thrombopenia of 86 G/L)). CRP increased to 352 mg/L. Chest X‐ray revealed pneumonia with bilateral pleural effusions. A diagnosis of toxic shock syndrome (TSS) was also made. He received fluid resuscitations with subsequent clinical stabilization. He also received clindamycin and intravenous human immunoglobulin (1 g/kg) for suspected TSS. He remained stable but febrile. Four days later, he presented with extremity desquamation and a rise in inflammatory markers. A repeat chest X‐ray showed bilateral pleural effusions with mediastinal widening, already present on the first X‐ray but not noticed. CT confirmed the findings linked to mediastinitis, prompting transfer to our tertiary center on Day 10. No mediastinal drainage was performed. A right‐sided pleural drain was placed, draining citrine fluid, and was removed after 4 days. Pleural fluid culture, 16S PCR, and next‐generation sequencing were negative.

MRI on Day 17 showed infiltration of the thymus and mediastinal lymph nodes without collection along with a reduction in pleural effusions, allowing the switch to oral amoxicillin (80 mg/kg/day) for a total antibiotic duration of 6 weeks. He was transferred back to the referring hospital 8 days after arrival at our institution and was discharged home 20 days after initial presentation. At the end of antibiotic therapy, he was asymptomatic and doing well.

No immunological workup was performed. It was not performed during the acute phase of hospitalization at our institution, and after transfer back to the referring hospital, we were not directly involved in subsequent decisions regarding this aspect of care.

#### 2.1.4. Case Report 4

The patient was a 10‐year‐old boy fully vaccinated with no significant past medical history. He presented to the hospital with progressive right shoulder pain for 5 days and fever of 38°C for 2 days, 1 week after a ski fall on the right shoulder without immediate posttraumatic pain. He also reported a self‐resolving upper respiratory tract infection 3 weeks earlier. On arrival, the patient was in good general condition with normal vital signs (temperature of 37.9°C and HR of 105/min). The rest of the clinical examination, including right shoulder assessment, was unremarkable.

Because of isolated shoulder pain, an osteoarticular infection was suspected. Laboratory tests showed a CRP of 128 mg/L and a WBC count of 12 × 10^9^/L. Chest and right shoulder X‐rays were normal. Shoulder ultrasound revealed a small joint effusion. The child was admitted for suspected osteoarticular infection and treated with IV amoxicillin–clavulanate (150 mg/kg/day). An MRI was performed on hospital Day 1, initially focused on the right shoulder, and then extended to the contralateral shoulder for comparison. MRI did not confirm an osteoarticular infection but incidentally revealed inflammatory changes in the mediastinal fat with compression of the brachiocephalic vein, without fluid collection, leading to the diagnosis of mediastinitis. A thoracic CT scan was subsequently performed to address the differential diagnosis of mediastinal lymphadenopathy and did not support an alternative diagnosis. Blood cultures, nasopharyngeal swabs for influenza, RSV, and SARS‐CoV‐2, and a GAS pharyngeal swab were all negative.

The patient recovered rapidly without complications. Fever resolved on hospital Day 2, inflammatory markers decreased, and right shoulder pain disappeared. He was discharged after 6 days and switched to oral amoxicillin–clavulanate (80 mg/kg/day in three doses) for a total antibiotic duration of 4 weeks. A follow‐up MRI carried out 34 days after discharge showed complete regression of the mediastinitis, and the patient was completely asymptomatic.

No immunological workup was performed, as the infection rapidly resolved following early treatment, and there were no additional clinical or anamnestic features suggestive of an underlying immune deficiency.

Table 1 summarizes and compares the clinical and microbiological data and evolution of the four cases.

**TABLE 1:** Summary of the four cases.

	Case 1	Case 2	Case 3	Case 4
Patient’s sex	Girl	Boy	Boy	Boy

Patient’s age	6 years	8 months	6 years	10 years

Clinical presentation	Febrile torticollis with odynophagia, and lower cervical pain	Fever, cough, and stridor with acute respiratory failure	Fever, “flu‐like” symptoms, scarlet fever‐like rash, abdominal pain, vomiting, and odynophagia	Fever and shoulder pain

Identified pathogen	*Streptococcus pyogenes* in pleural fluid detected by 16S rRNA PCR	None	Positive rapid group A *Streptococcus* test	None

Viral coinfection	None	*Adenovirus* and *rhino/enterovirus* in nasopharyngeal PCR	Varicella	None

Radiological findings	Retrosternal collection measuring approximately 75 × 6 × 14 mm	Several phlegmonous areas in the anterior mediastinum	Infiltration of the thymus and mediastinal lymph nodes without fluid collection	Upper mediastinum infiltration without fluid collection

Length of IV antibiotic therapy	7 days	28 days	17 days	6 days

Global length of antibiotic therapy	28 days	9 weeks	6 weeks	4 weeks

Other treatments	‐ NIV ‐ Pleural drain	‐ Adrenaline nebulization ‐ NIV ‐ Corticosteroids	‐ Fluid resuscitation ‐ Intravenous human immunoglobulin ‐ Pleural drain	None

Short‐term complications	Acute respiratory failure	‐ Acute respiratory failure ‐ Toxic‐like syndrome	Toxic shock syndrome	None

Outcome	Complete recovery	Complete recovery	Complete recovery	Complete recovery

Abbreviation: NIV, noninvasive ventilation.

## 3. Discussion

Our four pediatric cases of acute nontraumatic mediastinitis represent an important contribution to the pediatric literature, as all were managed successfully with conservative treatment alone. The patients aged 8 months and 10 years old, presented with varied clinical manifestations and severity, but all achieved complete recovery without sequelae. This series emphasizes the feasibility and safety of antibiotic‐based management in carefully selected patients with mediastinitis.

Recent studies have shown that pediatric nontraumatic mediastinitis is associated with lower mortality rate compared to adult cases [1]. Mortality rates in adults range from 15% to 40% [1] whereas pediatric mortality is reported below 10% [10]. This difference suggests fundamental biological and clinical distinctions between the two populations. Prado‐Calleros et al. performed a systematic review of DNM (936 patients) including predominantly adult cases with only 12 pediatric cases [17]. The overall mortality rate was 19.5% but increased to 63% (range 51%–86%) without surgical treatment [11]. Among the 12 pediatric cases reviewed, 11 were managed surgically and one conservatively [8, 15]. All 12 patients survived. Pharyngeal and odontogenic infections were identified as the most frequent origin of DNM [17].

Most cases of mediastinitis are traumatic, particularly following cardiothoracic surgery. Postsurgical mediastinitis differs from nontraumatic forms in several aspects: It is a nosocomial infection affecting patients with significant comorbidities, and its microbiology more frequently includes gram‐negative bacteria and polymicrobial infections, requiring broader‐spectrum antimicrobial coverage [20]. Accordingly, although mortality is lower in children than in adults, it remains substantially higher than in nontraumatic mediastinitis, reaching up to 27% [20, 21]. Standardized pediatric treatment guidelines are lacking; however, antibiotic therapy combined with surgical intervention is generally recommended [20, 21].

Although DNM is the most common form of acute nontraumatic mediastinitis, two older children in our series had no clearly identified source. Since retropharyngeal lymph nodes typically regress by around 5 years of age, alternative mechanisms should be considered in this age group. In Case 3, a cervical origin with DNM was favored by odynophagia and a positive pharyngeal swab, although hematogenous dissemination, possibly facilitated by recent varicella‐associated invasive GAS infection, remained possible. In Case 4, the source remained unknown; transient bacteremia could not be excluded, and no primary focus or adjacent structural abnormality was identified. MRI revealed inflammatory changes in the mediastinal fat, and thoracic CT excluded mediastinal lymphadenopathy, supporting the diagnosis of mediastinitis rather than reactive adenopathy.

The incidental diagnosis suggests that mild forms may occasionally go unrecognized, particularly when symptoms are nonspecific or antibiotics are started early, but does not permit conclusions about the true frequency of underdiagnosis. In children, diagnostic suspicion is limited by the absence of validated pediatric‐specific risk factors or predictors. Adult predisposing factors for DNM include poor dentition, diabetes, AIDS, and drug or alcohol abuse [22]. In an adult series, symptoms did not discriminate between uncomplicated deep neck infections and those complicated by mediastinal abscess; higher CRP levels and involvement of retropharyngeal or anterior visceral spaces were associated with mediastinitis [23]. Although most adult risk factors are of limited relevance in children and CRP is nonspecific, the anatomical location of the infection may help identify children at risk of mediastinal extension. However, pediatric data supporting this association are lacking, and no other validated pediatric‐specific predictors are currently available.

We reviewed 45 published pediatric cases of acute nontraumatic mediastinitis. A total of 41 (91%) of them were managed surgically, while, to our knowledge, only four were treated conservatively [1, 6–10, 15, 18, 24–28]. This contrasts sharply with our experience, where multidisciplinary discussions involving intensivists, surgeons, ENT specialists, and infectious diseases specialists led to conservative management in all four cases. The decision was primarily based on radiological findings: Cervical imaging revealed no drainable abscesses, and mediastinal extension was limited to phlegmonous inflammation without any organized collection requiring surgical drainage.

Our patients appear to have common features with 4 clearly documented pediatric cases of conservatively managed mediastinitis. Wright et al. reported a pediatric patient with retropharyngeal phlegmon showing slight extension into the superior mediastinum but lacking fluid collection. There were no bacterial cultures obtained. This patient was successfully managed with medical therapy alone (unknown duration of antibiotics), similar to our approach [15]. Lira et al. described a 5‐year‐old child with 3 mediastinal collections (the largest measuring 40 × 14 × 27 mm) [27]. *Streptococcus pyogenes* was retrieved from blood culture. Despite initial consideration for surgical drainage, a follow‐up CT scan after 4 days of intravenous antibiotics demonstrated regression of the mediastinal lesions. Given clinical stability and radiological improvement, surgical intervention was deferred. This case received a total of 4 weeks of intravenous antibiotic therapy, with complete recovery. Hadhud et al. described a 1‐year‐old girl with DNM complicated by bilateral pleural effusions and pericardial effusion [7]. Despite the severity, she was treated conservatively with bilateral pleural drains and prolonged antibiotic therapy for a total of 74 days (33 days intravenously). Blood and pleural cultures grew *S. pyogenes*. She achieved full clinical and radiological recovery without surgical mediastinal drainage. Finally, Clark et Lobo reported a 7‐year‐old boy with bacterial tracheitis complicated by pretracheal cellulitis and mediastinitis [28]. CT scan showed an inflammatory collection extending form the pretracheal region into the suprasternal region of the mediastinum measuring 2.7 cm in diameter. Serology was suggestive of a *Streptococcus pyogenes* infection. The patient was treated fully conservatively with 48 h of intravenous antibiotics and 12 days of oral antibiotics with complete recovery. Our series adds four cases to this limited literature, with antibiotic durations ranging from 3 to 9 weeks and intravenous therapy lasting 6–28 days. Like the previously reported cases, all our patients remained clinically stable or improved with medical management, despite significant complications such as pleural effusions (three patients) and respiratory failure (one patient). Pleural effusions were managed as pediatric parapneumonic effusions, with drainage guided by effusion size and respiratory impact; sepsis would also have prompted urgent drainage. IV‐to‐oral switch was individualized based on clinical improvement, absence of bacteremia or organized abscesses, and favorable radiological evolution, generally allowing early transition to oral therapy, except in Case 2, where persistent fever spikes and rash justified prolonged IV treatment. Unlike postsurgical mediastinitis, the absence of prosthetic material, nosocomial infection, or multidrug‐resistant pathogens supported earlier oral switch once patients improved clinically.

These observations suggest that, in clinically stable pediatric patients receiving appropriate antibiotic therapy and without well‐defined drainable collections, conservative management is a reasonable and effective approach. In contrast, our review identified that 35 of the 41 surgically treated patients had either an abscess or a fluid collection within the retropharyngeal space or mediastinum documented on imaging [1, 4, 6, 7, 9, 13–16].

The microbiology of pediatric nontraumatic mediastinitis is dominated by GAS or *Staphylococcus aureus* [6, 7]. In our case series, GAS infection was confirmed in Patient 1 (*Streptococcus pyogenes* detected by 16S rRNA PCR in pleural fluid) and strongly suspected in Patients 2 and 3. These three cases occurred between December 2022 and April 2023, a period marked by a surge in invasive streptococcal infections in Europe [29]. Patient 3 had varicella, a well‐established risk factor for invasive GAS infection. Moreover, the toxin‐like symptoms, prolonged inflammatory course, high degree of inflammation, and extremity desquamation observed in Patients 2 and 3 strongly support streptococcal etiology, even in the absence of microbiological confirmation. Interestingly, three previously reported pediatric cases of conservatively managed mediastinitis with documented microbiology were also associated with GAS infection [7, 27, 28]. GAS exhibits remarkable susceptibility to penicillin, with consistently low minimal inhibitory concentrations (MIC) and no significant resistance issues, unlike *Staphylococcus aureus,* with high risk of methicillin resistance in specific settings. If antibiotics are administered promptly, the strong susceptibility of GAS to beta‐lactams may allow these infections to be controlled directly without the need for surgical drainage. However, conservative management entails an inherent trade‐off: While avoiding an invasive procedure may reduce procedural morbidity, it also limits microbiological documentation. This approach should, therefore, be reserved for clinically stable patients in whom the clinical and epidemiological context does not suggest infection with multidrug‐resistant pathogens.

Our case series is limited by the low number of patients and the absence of surgically managed patients for comparison. We cannot directly assess differences in complications, hospitalization duration, or outcomes between conservative and surgical approaches. Some of our patients experienced severe complications (extension of the infection to the pleura and lung, toxic manifestations) and required prolonged antibiotic therapy (up to 9 weeks), reflecting the varying degrees of severity and different clinical evolution course of this infectious disease [30]. The conservatively managed cases reported by Hadhud et al., Lira et al., and Clark et Lobo provide useful context for our experience; Hadhud et al.’s patient received 74 days of total antibiotic therapy [7], Lira et al.’s patient 4 weeks of intravenous antibiotics [27], and Clark et Lobo’s patient 14 days of antibiotics [28]. When these published cases are considered with our own, the total duration of antibiotic therapy for conservatively managed pediatric mediastinitis ranges from 2 to 10.5 weeks. Whether surgical management would have shortened antibiotic therapy or reduced complications remains unknown. Moreover, as discussed above, the predominance of confirmed or suspected GAS in our series may have biased outcomes toward a favorable response to conservative treatment and may not apply to other etiologies.

Our case series and the limited published literature prompt consideration of which pediatric patients with mediastinitis may be safely managed without surgery. Based on the available data, potential selection criteria for conservative management may include radiological findings (absence of well‐defined, drainable fluid collections; phlegmonous inflammation without organized abscesses), clinical stability, absence of uncontrolled sepsis, no relevant comorbidities or known/suspected immunodeficiency, and favorable microbiological features (infection with highly susceptible organisms such as GAS), although this is often unknown at presentation. Conservative management should be considered only after multidisciplinary consensus and in settings where close monitoring and a low threshold for surgical intervention are ensured, including access to intensive care support, serial clinical assessments, repeated CT or MRI, and expertise from pediatric infectious disease, surgical, radiological, and intensive care teams. However, these criteria remain speculative and are derived from limited retrospective case reports with inherent selection bias. Additional data are necessary to identify reliable predictors of treatment success or failure, optimal antibiotic regimens, and to establish monitoring protocols (frequency and modality of imaging, clinical thresholds for escalation).

In conclusion, pediatric acute nontraumatic mediastinitis is a rare but severe infection, associated with significant complications. Management remains poorly defined in the pediatric population and most reported cases have required surgical intervention. Our case series demonstrates that pediatric acute nontraumatic mediastinitis can be successfully treated with conservative, antibiotic‐based management in carefully selected patients, particularly those without organized fluid collections. However, the extremely limited number of conservatively managed cases precludes definitive conclusions. In addition, our results should be interpreted within the context of the regional epidemiological behavior of *Streptococcus pyogenes*. In other epidemiological settings and resistance patterns, broader antibiotics may be required. Further studies in pediatric populations are essential to establish evidence‐based indications and optimal strategies for nonsurgical management.

## Funding

The authors have nothing to report.

Open access publishing facilitated by Universite de Lausanne, as part of the Wiley ‐ Universite de Lausanne agreement via the Consortium Of Swiss Academic Libraries.

## Ethics Statement

The Ethics Committee of the Canton of Vaud (CER‐VD) waives the authorization requirement for case series involving fewer than five patients.

## Consent

Parents of the four patients gave written consent for the publication of the anonymized clinical data.

## Conflicts of Interest

The authors declare no conflicts of interest.

## Data Availability

The data that support the findings of this study are available on request from the corresponding author. The data are not publicly available due to privacy or ethical restrictions.

## References

[1] Wilson C. D. , Kennedy K. , Wood J. W. et al., Retrospective Review of Management and Outcomes of Pediatric Descending Mediastinitis, Otolaryngology–Head and Neck Surgery. (2016) 155, no. 1, 155–159, 10.1177/0194599816634636. 26932964

[2] Kumar N. , Adult Retropharyngeal Abscess: A Retrospective Case Series, An International Journal of Otorhinolaryngology Clinics. (2015) 7, no. 2, 100–103, 10.5005/jp-journals-10003-1202.

[3] Jain H. , Hohman M. H. , and Sinha V. , Retropharyngeal Abscess, 2026, StatPearls Publishing, Treasure Island (FL). 28722903

[4] Oliphant M. , Wiot J. F. , and Whalen J. P. , The Cervicothoracic Continuum, Radiology, 10.1148/120.2.257. 935471

[5] Smith J. K. , Armao D. M. , Specter B. B. , and Castillo M. , Danger Space Infection: Infection of the Neck Leading to Descending Necrotizing Mediastinitis, Emergency Radiology. (1999) 6, no. 3, 129–132, 10.1007/s101400050038.

[6] Cox J. , Byrne A. T. , Harty S. , McGuinness J. , Russell J. , and Geoghegan S. , Descending Necrotising Mediastinitis, BMJ Case Reports CP. (2025) 18, no. 3, 10.1136/bcr-2024-264342. 40032574

[7] Hadhud M. , Sneineh M. A. , Averbuch D. , and Breuer O. , The Hidden Highway: a Forgotten Complication of Oropharyngeal Infection in Children, Pediatric Pulmonology. (2024) 59, no. 3, 776–778, 10.1002/ppul.26807. 38088229

[8] Shah R. K. , Chun R. , and Choi S. S. , Mediastinitis in Infants From Deep Neck Space Infections, Otolaryngology–Head and Neck Surgery. (2009) 140, no. 6, 936–938, 10.1016/j.otohns.2009.02.032. 19467419

[9] Marques D. L. , Rato C. , Miguéis A. , and Miguéis J. , Descending Necrotising Mediastinitis: A Rare Entity in Children, BMJ Case Reports CP. (2024) 17, no. 3, 10.1136/bcr-2023-258304. PMC10921524 38453221

[10] Weiner K. A. S. , Rieger C. C. , Wohl D. L. , and Harley E. H. , Retropharyngeal Abscess With Mediastinal Extension: A Case Series and Review of the Literature, Ear, Nose & Throat Journal. (2023) 102, no. 9, 580–583, 10.1177/01455613231178975. 37309202

[11] Zineb J. , Moboula C. , Meryem N. , Abdallah F. , Kamilia C. , and Souhail B. , *Staphylococcus aureus* Bacteremia with a Mediastinal Abscess in a 9-month-old Infant: a Case Report and Literature Review, The Pan African Medical Journal. (2023) 44, 10.11604/pamj.2023.44.173.38638. PMC10349636 37455893

[12] Deu-Martín M. , Saez-Barba M. , López Sanz I. , Alcaraz Peñarrocha R. , Romero Vielva L. , and Solé Montserrat J. , Factores De Riesgo De Mortalidad En La Mediastinitis Necrosante Descendente, Archivos de Bronconeumología. (2010) 46, 182–187, 10.1016/j.arbres.2010.01.008. 20227809

[13] Adil E. , Tarshish Y. , Roberson D. , Jang J. , Licameli G. , and Kenna M. , The Public Health Impact of Pediatric Deep Neck Space Infections, Otolaryngology–Head and Neck Surgery. (2015) 153, no. 6, 1036–1041, 10.1177/0194599815606412. 26408562

[14] Patigaroo S. A. , Patigaroo F. A. , Mehfooz N. , Khan N. A. , Kirmani M. H. , and Shakeel , Pediatric Deep Neck Space Abscesses: a Prospective Observational Study, Emergency Medicine: Open Access, 2, 1–6, 10.4172/2165-7548.1000117.

[15] Wright C. T. , Stocks R. M. S. , Armstrong D. L. , Arnold S. R. , and Gould H. J. , Pediatric Mediastinitis as a Complication of Methicillin-Resistant *Staphylococcus aureus* Retropharyngeal Abscess, Archives of Otolaryngology-Head and Neck Surgery. (2008) 134, no. 4, 408–413, 10.1001/archotol.134.4.408. 18427007

[16] Dueñas J. , García-Menor E. , de la Rosa I. I. , Granados A. , Antón M. , and Pérez-Navero J. L. , Descending Necrotizing Mediastinitis in Early Childhood: Favorable Outcome After Aggressive Treatment, Pediatric Critical Care Medicine. (2003) 4, 476–479, 10.1097/01.PCC.0000074277.07221.38. 14525646

[17] Prado–Calleros H. M. , Jiménez–Fuentes E. , and Jiménez–Escobar I. , Descending Necrotizing Mediastinitis: Systematic Review on Its Treatment in the Last 6 years, 75 Years After Its Description, Head & Neck. (2016) 38, no. S1, E2275–E2283, 10.1002/hed.24183. 26829352

[18] Hasjim B. J. , Ostowari A. , Holmes W. N. et al., Methicillin-Resistant *Staphylococcus aureus* Descending Necrotizing Mediastinitis in an Infant, Journal of Pediatric Surgery Case Reports. (2023) 89, 10.1016/j.epsc.2022.102572.

[19] Moola S. , Munn Z. , Tufanaru C. et al., Aromataris E. and Munn Z. , Chapter 7: Systematic Reviews of Etiology and Risk, JBI Manual for Evidence Synthesis, 2020, JBI, https://synthesismanual.jbi.global.

[20] Mangukia C. V. , Agarwal S. , Satyarthy S. , Datt V. , and Satsangi D. , Mediastinitis Following Pediatric Cardiac Surgery, Journal of Cardiac Surgery. (2014) 29, no. 1, 74–82, 10.1111/jocs.12243. 24267786

[21] Bernheim S. , Chikhi S. , Foufa I. et al., Postoperative Mediastinitis in Pediatric Cardiac Surgery: Clinical Characteristics, Management and Risk Factors for Mortality, The Pediatric Infectious Disease Journal. (2025) 44, no. 11, 1025–1029, 10.1097/INF.0000000000004882. 40472255

[22] Hu C.-Y. , Lien K.-H. , Chen S.-L. , and Chan K.-C. , Risk Factors of Descending Necrotizing Mediastinitis in Deep Neck Abscesses, Medicina. (2022) 58, no. 12, 10.3390/medicina58121758. PMC9788205 36556959

[23] Taylor M. , Patel H. , Khwaja S. , and Rammohan K. , Descending Cervical Mediastinitis: the Multidisciplinary Surgical Approach, European Archives of Oto-Rhino-Laryngology. (2019) 276, no. 7, 2075–2079, 10.1007/s00405-019-05471-z. 31093735

[24] Sharma K. , Silver F. , Tengsupakul S. , Hartin C. , Wong K. , and Estrada B. , 617: Mediastinitis, Empyema, and Chest Wall Infiltration From the Retropharyngeal Abscess: A Rare Event, Critical Care Medicine. (2025) 53, no. 1, 10.1097/01.ccm.0001101132.87183.1c.

[25] Veronese P. , Cella S. , Giacometti A. et al., Invasive Streptococcus intermedius Infections in Children: Two Cases From a Pediatric Infectious Diseases Unit in Italy, Pathogens. (2024) 13, no. 12, 10.3390/pathogens13121099. PMC11728730 39770358

[26] Thomas A. , Adam S. , Goussard P. , Venkatakrishna S. S. B. , Andronikou S. , and Grobbelaar J. , Retropharyngeal Abscess Complicated by Mediastinitis in Infants, Respiration. (2024) 103, no. 10, 651–659, 10.1159/000540525. 39084200

[27] Lira J. , Santos J. , Capela M. , Rodrigues J. , Cunha O. , and Carvalho I. , Descending Mediastinitis in a Child: Diagnostic Challenges and a Different Treatment Approach, Clinics and Practice. (2021) 11, no. 3, 505–508, 10.3390/clinpract11030066. 34449574 PMC8395469

[28] Clark M. T. and Lobo M. , Letters to the Editor, Journal of Paediatrics and Child Health. (2008) 44, no. 11, 676–677, 10.1111/j.1440-1754.2008.01400.x. 19012645

[29] Lassoued Y. , Assad Z. , Ouldali N. et al., Unexpected Increase in Invasive Group A Streptococcal Infections in Children After Respiratory Viruses Outbreak in France: a 15-Year Time-Series Analysis, Open Forum Infectious Diseases. (2023) 10, no. 5, 10.1093/ofid/ofad188. PMC10167988 37180594

[30] McMullan B. J. , Andresen D. , Blyth C. C. et al., Antibiotic Duration and Timing of the Switch From Intravenous to Oral Route for Bacterial Infections in Children: Systematic Review and Guidelines, The Lancet Infectious Diseases. (2016) 16, no. 8, e139–e152, 10.1016/S1473-3099(16)30024-X. 27321363

